# Selective correlation of hippocampal volumes with WADA memory scores in mesial temporal sclerosis patients

**DOI:** 10.3389/fneur.2025.1507846

**Published:** 2025-01-24

**Authors:** Lourdes Khalife, Wassim Nasreddine, Fatima Jaafar, Huda Abboodi, Karim Nasreddine, Ahmad Beydoun

**Affiliations:** ^1^Department of Neurology, American University of Beirut Medical Center, Beirut, Lebanon; ^2^Maroun Semaan Faculty of Engineering and Architecture, American University of Beirut, Beirut, Lebanon

**Keywords:** WADA test, hippocampal volumes, mesial temporal sclerosis, epilepsy, magnetic resonance imaging

## Abstract

**Objective:**

The WADA test is used to determine cerebral language dominance and assess the risk of postoperative amnesia following mesial temporal lobe resection. This study aims to explore the correlation between automated measures of hippocampal volume and WADA memory scores and to evaluate whether these volumetric measurements can reliably predict WADA memory scores.

**Methods:**

This study included patients who underwent a comprehensive presurgical assessment along with bilateral WADA testing. Hippocampal volumes were measured from high-resolution brain MRIs using automated software (volBrain), which were harmonized and normalized to whole brain volume. These harmonized and normalized volumes were then correlated with ipsilateral WADA memory scores and stratified according to brain MRI findings. A similar analysis was conducted between hippocampal volume asymmetry and WADA memory score asymmetry (WMA). A Receiver Operating Characteristic (ROC) curve was generated to compare the sensitivity and specificity in predicting successful WADA outcomes based on ipsilateral harmonized normalized hippocampal volumes.

**Results:**

In patients with mesial temporal sclerosis (MTS), significant positive correlations were found between harmonized normalized hippocampal volumes and ipsilateral WADA memory scores, as well as between harmonized hippocampal volume asymmetries and WMA. However, no significant correlations were found in patients with epileptogenic lesions other than MTS or those with normal brain MRIs. A harmonized normalized hippocampal volume threshold of ≥ 28.94 units was identified as a predictor of a WADA memory score exceeding 50% following contralateral carotid artery injection, with a sensitivity of 62.1% and a specificity of 100%.

**Significance:**

This study indicates that hippocampal volumetry could potentially serve as an alternative to the WADA test in patients with MTS. Conversely, in individuals with normal MRI results or other types of epileptogenic lesions, hippocampal volumetry does not reliably predict memory deficits, necessitating the use of the WADA test or functional MRI for planning resections of mesial temporal structures in the dominant hemisphere.

## 1 Introduction

The WADA test, also known as the intraarterial amobarbital procedure, was initially introduced by Juhn Wada in the mid-twentieth century as a means of assessing cerebral language dominance (1). It later evolved into a crucial tool for estimating the risk of postoperative memory deficits in patients scheduled for a mesial temporal lobe resection (2, 3). This procedure selectively inactivates a single hemisphere by injecting an anesthetic agent into the carotid artery. By doing so, it provides a means to evaluate the functional contribution of mesial temporal lobe structures involved in language and memory processing. When both carotid arteries are injected, the functional adequacy of the hemisphere ipsilateral to the epileptogenic zone and the functional reserve capacity of the contralateral hemisphere can be assessed. This dual injection approach allows for the evaluation of memory function asymmetry which helps in predicting seizure laterality and postoperative seizure freedom (4, 5).

Despite its widespread acceptance due to its low complication rates (6), the invasive nature of the WADA test has driven researchers to explore less invasive alternatives. Functional MRI (fMRI) has emerged as a promising alternative and has nearly supplanted the WADA test for language lateralization. However, concerns about the reliability of fMRI for memory assessment have been raised due to imperfect agreement between fMRI and the WADA test, the lack of standardized protocol and the required patient cooperation, as reported by various studies (7–10).

An alternative avenue pursued by researchers to obviate the need for the WADA test is by assessing surrogate markers that could predict memory function, such as hippocampal volume measurements (11–13). Some of these studies identified associations between absolute memory scores and absolute hippocampal volumes (13), while others found significant correlations between left–right hippocampal volume asymmetry and interhemispheric WADA memory score asymmetry (WMA) (11, 12). It is worth noting that the most recent of these investigations was conducted 20 years ago, and that these studies had several limitations. These included volumetric measurements of the hippocampi assessed on an oblique coronal series, with the volume calculated by multiplying the total number of pixels by the size of the pixel and by slice thickness. In addition, normalization of the hippocampal volume based on total brain volume was not performed, and some studies did not stratify patients based on brain MRI findings, which could influence the interpretation of results.

Recent advances in quantitative neuroimaging have led to the development of automated segmentation protocols, enabling more accurate measurements of hippocampal volumetry and faster volumetric assessments. This study aims to investigate the correlation between automated hippocampal volumetry measures and WADA memory scores with patients stratified based on brain MRI findings. The goal is to evaluate whether automated hippocampal volumetry can reliably predict the WADA memory scores, offering a less invasive approach for presurgical evaluation.

## 2 Materials and methods

### 2.1 Study population

This retrospective study included all patients who were evaluated at the American University of Beirut Medical Center (AUBMC) between January 2012 and December 2023 and underwent a comprehensive presurgical assessment along with bilateral WADA testing. Patients who underwent a unilateral WADA testing or had the test solely performed for language lateralization only without memory assessment were excluded from the study.

The presurgical workup typically included a detailed description of the seizure semiologies, physical examination, long-term video electroencephalography (EEG) monitoring, and an epilepsy protocol brain MRI. Additionally, subtraction ictal and inter-ictal single-photon emission computed tomography (SPECT) co-registered to MRI (SISCOM) and positron emission tomography (PET) were obtained to localize the epileptogenic zone, particularly in cases with normal MRI findings or non-localizing EEG results. The results of these tests were reviewed during a multidisciplinary refractory epilepsy conference to determine if the patient was a surgical candidate, the type and extent of resection needed, or whether additional tests such as invasive EEG monitoring or a WADA test were required prior to the surgery. Typically, we performed a WADA test on all patients with a planned resection of the mesial structures on the dominant hemisphere, or in patients with bilateral mesial temporal sclerosis (MTS).

### 2.2 The WADA test

The procedural details followed at AUBMC were previously outlined and are based on established protocols from Montreal and Boston Children’s Hospitals (14). At our institution, we always perform bilateral WADA testing, as research suggests that insights gained from this procedure can help predict the likelihood of achieving postoperative seizure freedom and assess the functional integrity of the mesial structures planned for resection. Studies have shown that patients demonstrating expected memory asymmetry on the WADA test have an increased probability of experiencing seizure freedom following surgery (2, 4, 15).

During the procedure, conducted under continuous EEG monitoring, the internal carotid artery was selectively catheterized via the femoral artery route, and the arteriogram was evaluated for cross-filling. Anesthetic agents (propofol or etomidate) were then administered to induce contralateral hemiplegia. Muscle strength was continuously monitored, with additional doses of anesthetic given as needed to maintain muscle weakness throughout the procedure. In our protocol, we first catheterized the internal carotid artery on the hemisphere scheduled for resection, allowing memory assessment on the non-surgical hemisphere. Subsequently, we anesthetized the hemisphere not planned for resection. Both hemispheres were assessed on the same day for all patients.

While one hemisphere was anesthetized, the patient was presented with two figures, two words, and eight objects. Following recovery of arm weakness and return of the EEG to baseline, the patient was asked to recall the previously presented words and figures from a list. Additionally, the patient was shown 24 objects, and tasked with identifying the 8 objects introduced during the test. The WADA memory score was derived from object recall and calculated out of a total of 8 points as follows: for every omission, one point was deducted, whereas two commissions resulted in a subtraction of 1 point. Subsequently, the overall score was normalized to a percentage with a score of 2 equating to 25% and a score of 8 amounting to 100%. At our institution, to mitigate the risk of substantial memory impairment after hippocampal resection, we required a WADA memory score of at least 4 to proceed with the surgery (16). Notably, the WADA memory score for the right hemisphere pertained to the score attained after injection of the left hemisphere, and vice versa.

### 2.3 Brain MRI image acquisition and volumetric analyses

Brain MRIs were acquired on two different scanners from the same manufacturer (Ingenia; Phillips Healthcare): 25 scans were performed on a 3 Tesla machine, and 23 scans on a 1.5 Tesla machine. The imaging-acquisition protocol consisted of 3D T1 (1 mm slice thickness) and 3D fast fluid-attenuated inversion recovery (FLAIR; 0.9- or 1-mm slice thickness) of the whole brain with multiplanar reconstruction, axial and coronal inversion recovery (2 mm slice thickness), axial T2 TSE and T2 FFE (4 mm slide thickness) and axial diffusion weighted images (4–5 mm slice thickness). The 3D images were obtained with no interslice gap. The MRIs were reviewed by an experienced neuroradiologist, who was blinded to the clinical data, and categorized into three groups: evidence of MTS, presence of epileptogenic lesions other than MTS, and normal MRI findings.

Volumetric analyses of the brain MRIs were performed using the VolBrain software,^1^ an automated online brain volumetry system (17, 18). We dcm2nii software (part of the MRICRON software) was used to convert T1-weighted images from DICOM to NIFTY format and anonymize the data. Whole-brain segmentation was conducted using an automated technique that separates tissue from CSF through thresholding and iterative erosions and dilations (17, 18). Before segmentation, preprocessing steps were applied to enhance input MRI quality and align images to a standardized geometric and intensity space, consistent with manually labeled training templates. These steps included spatially adaptive non-local means denoising, inhomogeneity corrections (rough and fine), affine registration to MNI space, intensity normalization, and extraction of intracranial cavities. Tissue classification, hemisphere segmentation, and subcortical structure segmentation were performed using precision-driven algorithms. These processes ensure robust and reliable volumetric analyses, including measurements of total intracranial volume, hemispheric volumes (gray matter, white matter, and cerebrospinal fluid), and specific cortical and subcortical structures (17, 19). For this study, the right and left hippocampal volumes were normalized by dividing them by the whole brain volume with ventricles extracted, as this normalized volume was previously shown to best correlate with memory performance (20). To minimize the impact of scanner variability, including differences in scanner type and field strength, the normalized volumes of the right and left hippocampi were harmonized using Neuroharmony tool.^2^ The harmonization process incorporated age, gender, scanner type, and field strength as covariates (21). Since expressing the harmonized normalized hippocampal volume as a percentage of total gray and white matter volumes yielded very small values (e.g., 0.2% of total gray and white matter volume), these hippocampal volumes were multiplied by 100 to simplify the communication of results (i.e., 0.20% normalized volume became 20 adjusted normalized units).

### 2.4 Data collection and analysis

The data collected for this study included patient gender, age at MRI acquisition, WADA memory scores, and harmonized normalized hippocampal volumes.

To assess WMA, we used a simple score calculated by subtracting the right hemisphere memory score from the left hemisphere memory score (left WADA memory score – right WADA memory score) (13). A positive WMA indicated a better memory score in the left hemisphere compared to the right hemisphere, whereas a negative score indicated the opposite, with the right hemisphere memory score surpassing the left hemisphere memory score.

For the volumetric analysis, we defined hippocampal volume asymmetry using a formula previously reported: (harmonized normalized left hippocampal volume – harmonized normalized right hippocampal volume) (11).

### 2.5 Statistical analysis

We reported the WADA memory scores and hippocampal volumes as means and standard deviations, stratified by the MRI groups.

A linear correlation analysis was conducted to examine the relationship between harmonized normalized hippocampal volumes and ipsilateral WADA memory scores, as well as between WMA and harmonized normalized hippocampal volume asymmetries. These correlations were performed separately within each MRI group and reported as Spearman correlation coefficients. To account for multiple comparisons (total of four), a Bonferroni correction was applied, with significance set at *p* < 0.0125.

Subsequently, a Receiver Operating Characteristic (ROC) curve was generated to compare sensitivity versus specificity for predicting successful WADA outcomes (defined as a WADA memory score of 4 or higher) based on ipsilateral harmonized normalized hippocampal volume. The Youden index was used to determine the optimal threshold between sensitivity and specificity.

### 2.6 Ethical approval and patient consent

This study was approved by the Institutional Review Board of AUBMC, and all enrolled patients provided informed consent.

## 3 Results

### 3.1 Demographics

Forty-eight patients (23 males and 25 females), with a mean age of 25.5 years ±8.9 years, underwent bilateral WADA testing and were included in the study. Among these patients, 21 had evidence of MTS on their MRI scans, 14 had epileptogenic lesions other than MTS, and 13 had normal brain MRIs.

Of the 21 patients with MTS, 16 had evidence of left MTS, three had right MTS, and two manifested bilateral MTS. Notably, two out of the three patients with right MTS were left-handed, while the third experienced ictal onset from both the right and left temporal lobes. The thirteen patients with normal brain MRIs underwent WADA testing based on the presurgical work-up indicating an ictal onset zone in the left temporal lobe. Regarding the MRI findings of the 14 patients with epileptogenic lesions other than MTS, these included low-grade tumors or cortical dysplasia involving the left mesial temporal structures in 13 patients. The remaining patient was left-handed with imaging evidence of right temporo-parietal encephalomalacia.

### 3.2 WADA memory score according to brain MRI findings

**Figure 1:**
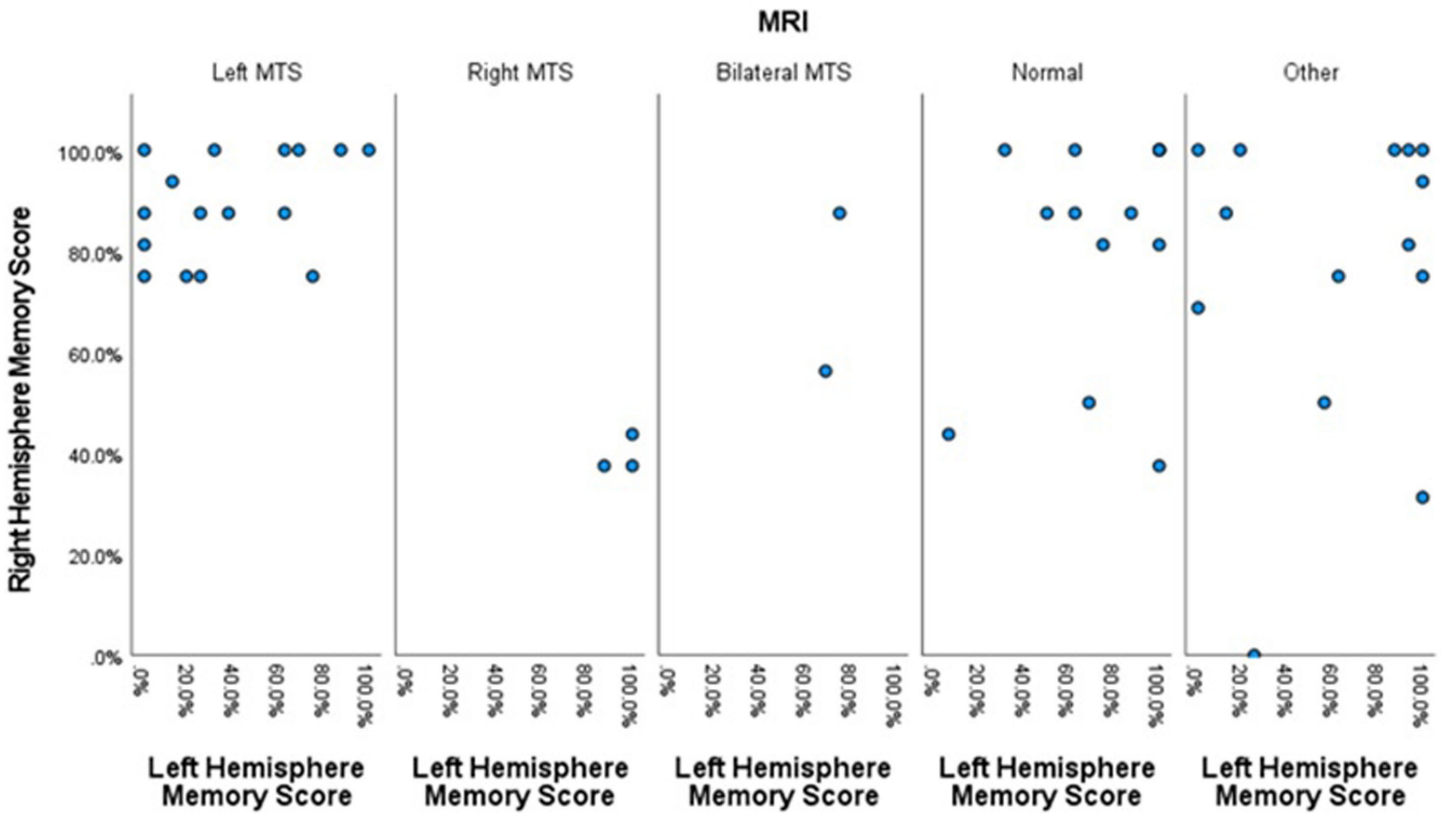
Scatter plot showing the left and right hemisphere memory scores for each patient across the five MRI groups.

The means of WADA memory scores for the patients with evidence of left MTS, right MTS, bilateral MTS, normal MRIs, and other epileptogenic lesions, are depicted in Figure 1.

Left hemisphere memory scores were lowest in patients with left MTS (mean = 37.9%, SD = 33.6%) and highest in those with right MTS (mean = 95.8%, SD = 7.2%).

The right hemisphere memory scores were lowest in patients with right MTS (mean = 39.6%, SD = 3.6%), while higher scores were noted in those with left MTS (mean = 89.1%, SD = 10.3%).

Patients with right MTS showed a large positive WMA (mean = 56.3%, SD = 6.3%), whereas those with left MTS exhibited a large negative WMA (mean = −51.2%, SD = 31.1%). Patients with bilateral MTS, normal brain MRI, and other epileptogenic lesions had substantially lower WMAs, with means of 0% ± 17.6, −8.7% ± 33.2%, and −15.2% ± 48.3%, respectively.

In one patient with bilateral MTS, the WADA memory scores of the left and right hemispheres were 75.00 and 87.50%, respectively. The corresponding scores for the other patients were 68.75 and 56.25%, respectively (Supplementary Table 1).

The absolute WMA differed significantly between patients with MTS (52.0%) and the combined group of patients with either normal MRIs or epileptogenic lesions other than MTS (30.1%) (*p* = 0.02).

In all patients with a normal MRI or with epileptogenic lesions other than MTS, WMA, when present by more than 20%, corresponded with the side of the lesion (in cases of patients with epileptogenic lesions other than MTS) or the side of the ictal onset zone (in cases of patients with normal MRI).

### 3.3 Hippocampal volume according to brain MRI findings

**Figure 2:**
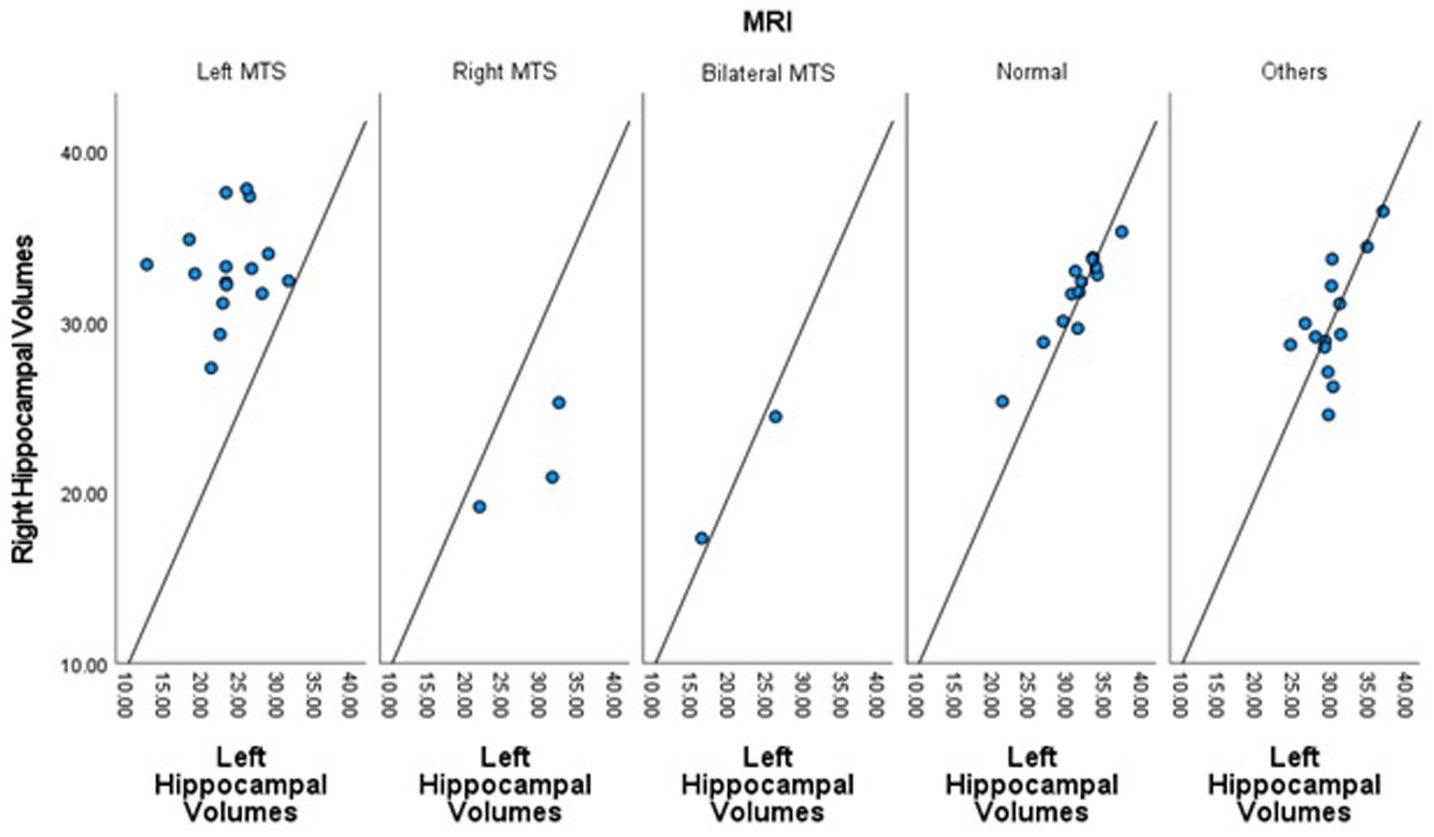
Scatter plot showing the harmonized normalized left and right hippocampal volumes for each patient across the five MRI groups.

The absolute and harmonized normalized hippocampal volumes, stratified by MRI findings are depicted in Figure 2 and Table 1.

**Table 1:** Hippocampal volumes and harmonized normalized hippocampal volumes across patient groups.

	Mean ± SE of hippocampal volumes (mm^3^)		Mean ± SE of harmonized normalized hippocampal volume	
	*L*	*R*	*L*	*R*
Left MTS (*N* = 16)	2,580 ± 130	3,740 ± 120	23.33 ± 1.14	33.09 ± 0.71
Right MTS(*N* = 3)	3,530 ± 180	2,690 ± 30	28.44 ± 3.39	21.75 ± 1.81
Bilateral MTS (*N* = 2)	2,420 ± 40	2,320 ± 95	21.09 ± 4.92	20.86 ± 3.55
Normal (*N* = 13)	3,790 ± 100	3,860 ± 90	31.02 ± 1.08	31.57 ± 0.72
Other epileptogenic lesions (*N* = 14)	3,490 ± 130	3,450 ± 150	29.93 ± 0.82	29.94 ± 0.87

This table summarizes the mean ± standard error (SE) of raw hippocampal volumes (in mm^3^) and harmonized normalized hippocampal volumes (arbitrary units) for the left (L) and right (R) hemispheres across different patient groups: Left mesial temporal sclerosis (Left MTS), Right mesial temporal sclerosis (Right MTS), Bilateral mesial temporal sclerosis (Bilateral MTS), normal controls, and patients with other epileptogenic lesions.

The lowest harmonized left hippocampal volumes were found in patients with bilateral and left MTS, with means ± SD of 21.09 ± 6.96 and 23.33 ± 4.55, respectively.

For the harmonized right hippocampal volumes, the lowest means ± SD were observed in bilateral and right MTS at 20.86 ± 5.02 and 21.75 ± 3.13, respectively (Figure 2).

Patients with evidence of right MTS exhibited a positive harmonized hippocampal volume asymmetry value (6.69 ± 4.00), while those with left MTS had a negative asymmetry (−9.75 ± 5.05).

There was a significant difference in harmonized hippocampal volume asymmetry between patients with MTS (8.52 units) and those with normal MRIs or other epileptogenic lesions (1.73 units) (*p* < 0.001).

### 3.4 Correlations between harmonized normalized hippocampal volumes and WADA memory scores

**Figure 3:**
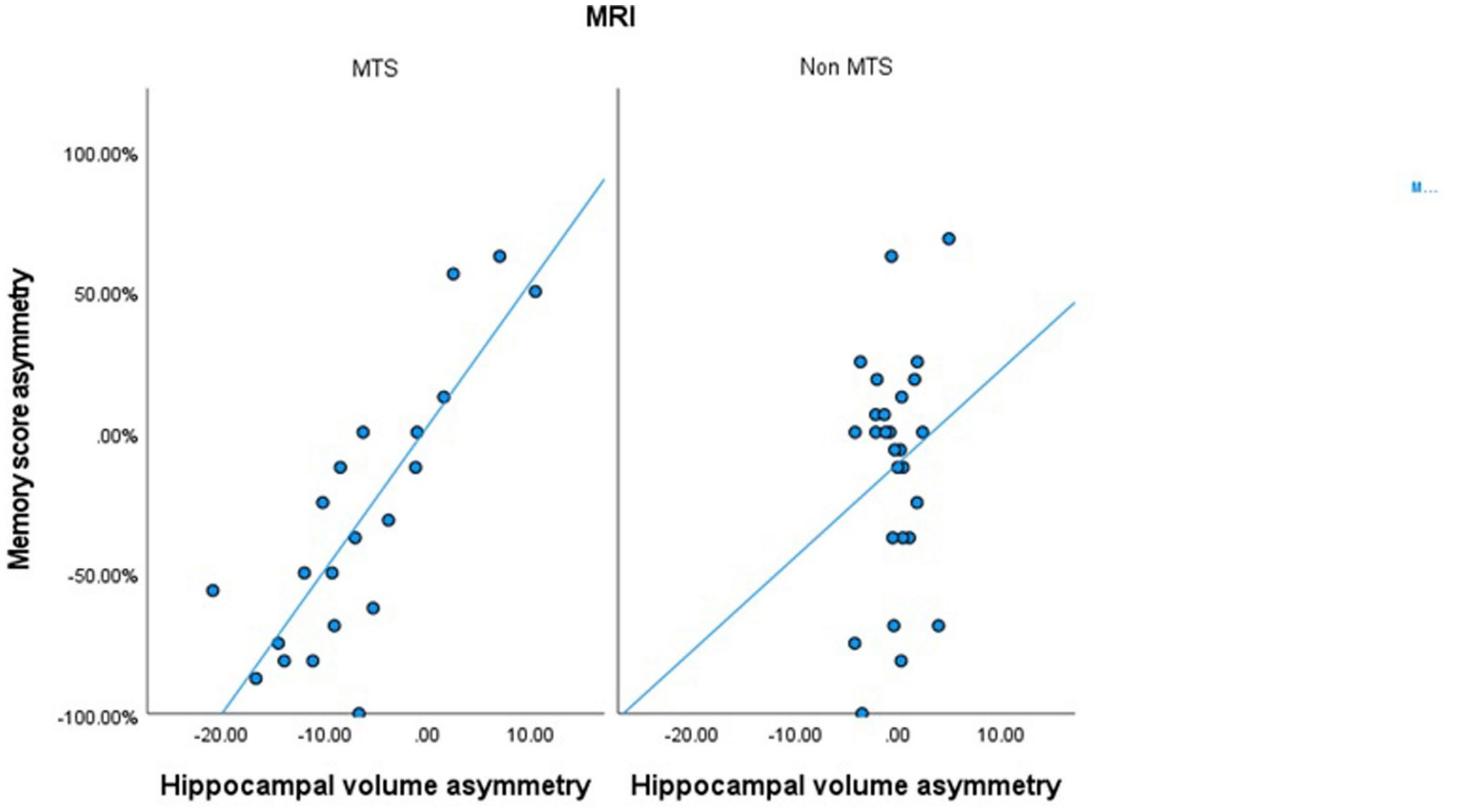
Scatter plot correlating the harmonized hippocampal volumes asymmetry and the memory score asymmetry in patients with MTS and in patients with normal MRIs or other epileptogenic lesions.

In patients with MTS, a significant correlation was observed between ipsilateral WADA memory scores and the ipsilateral harmonized normalized hippocampal volumes (*R* = 0.54, *p* < 0.001) (Table 2). Similarly, a significant correlation was found between WMA and harmonized hippocampal volume asymmetries (*R* = 0.774, *p* < 0.001) (Table 2; Figure 3). In all MTS patients, the WMA was concordant with the atrophic side (i.e., WADA memory scores were worse on the side of the MTS).

**Table 2:** Correlation between WADA memory scores and harmonized normalized hippocampal volume measurements (Significant *p*-value set at ≤0.0125 after Bonferroni correction).

MRI	Volumes	Scores	*R*	*p*-value
MTS	Hippocampus	Ipsilateral memory scores	0.540*	< 0.001
	Hippocampus asymmetry	Memory scores asymmetry	0.774*	< 0.001
Normal MRI or other epileptogenic lesions	Hippocampus	Ipsilateral memory scores	−0.131	0.344
	Hippocampus asymmetry	Memory scores asymmetry	0.019	0.926

The asterisk (*) next to the R values indicates significant positive correlations between the hippocampus volumes and the memory scores in patients with MTS.

On the other hand, in patients with a normal brain MRI or with other epileptogenic lesions, no significant correlation was observed between harmonized normalized hippocampal volumes and ipsilateral WADA memory scores, nor between hippocampal volume asymmetries and WMA (Table 2; Figure 3).

### 3.5 Failure of the WADA test

Among the 21 patients diagnosed with MTS who underwent 42 WADA memory tests, 13 patients failed the test (scoring less than 50%) after a unilateral injection. All these failures occurred when testing memory on the side of the atrophic hippocampus. The mean harmonized normalized hippocampal volume on the atrophic side for these 13 patients was 22.36 units (median 23.01 units), compared to an average of 29.29 units (median 31.61 units) on the 29 hippocampi that passed the WADA memory test.

Of the 13 patients with a normal MRI, 6 patients failed the WADA memory test after a unilateral injection. All these failures occurred when testing memory on the side concordant with the ictal onset zone. The mean harmonized normalized hippocampal volume on that side for these 6 patients was 33.16 units (median 33.17 units), compared to an average of 30.74 units (median 31.28 units) on the 20 hippocampi that passed the WADA memory test.

Out of the 14 patients with an epileptogenic lesion on MRI, 8 patients failed the WADA memory test after a unilateral injection. All these failures occurred when testing memory on the side of the epileptogenic lesion. The mean harmonized normalized hippocampal volume on that side for these 8 patients was 29.06 units (median 29.66 units), compared to an average of 30.29 units (median 29.45 units) on the 20 hippocampi that passed the WADA memory test.

### 3.6 Receiver operating characteristic curve in MTS patients

**Figure 4:**
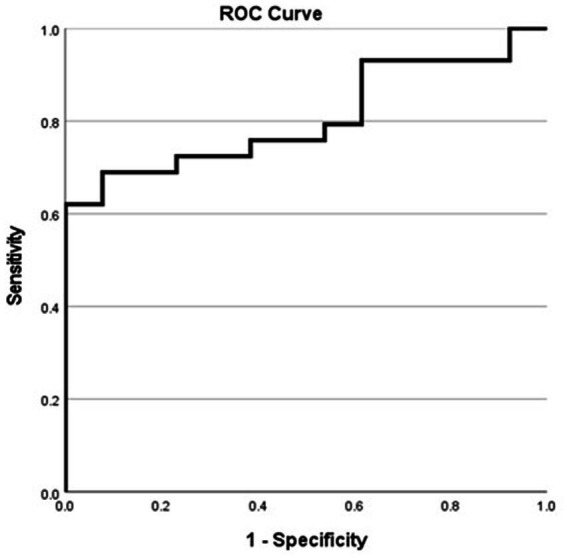
Receiver operating characteristic curve for MTS patients showing the sensitivity and specificity of various harmonized normalized hippocampal volumes in predicting successful WADA outcomes (defined as a WADA memory score of 50% or higher).

In patients with MTS, the ROC analysis yielded an area under the curve (AUC) of 0.806 (95% confidence interval, 0.67–0.93) (Figure 4). According to the Youden index, a hippocampal volume ≥ 28.94 units showed the highest discriminatory power in identifying patients who passed the WADA memory test, with a sensitivity of 62.1%, specificity of 100%, positive predictive value (PPV) of 100%, negative predictive value (NPV) of 54.2%, and an overall accuracy of 73.8%. The confusion matrix, specific to patients with MTS, further illustrates the diagnostic metrics (Table 3).

**Table 3:** Performance metrics for WADA memory test predictions based on brain volume thresholds.

	Passed WADA memory test	Failed WADA memory test	
Volume ≥ 28.94 units	TP =18	FP = 0	PPV = 100%
Volume < 28.94 units	FN = 11	TN =13	NPV = 54.2%
	Sensitivity = 62.1%	Specificity = 100%	Accuracy = 73.8%

This table summarizes the diagnostic performance metrics for predicting WADA memory test outcomes using a hippocampal volume threshold of 28.94 units. The table includes true positives (TP), false positives (FP), false negatives (FN), and true negatives (TN) along with the positive predictive value (PPV), negative predictive value (NPV), sensitivity, specificity, and overall accuracy.

## 4 Discussion

In our study, we found a significant positive correlation between harmonized normalized hippocampal volumes and ipsilateral memory scores in patients with MTS, indicating a strong association between hippocampal structural integrity and memory function. Moreover, our findings revealed that the harmonized hippocampal volumes asymmetry in MTS patients correlated with the asymmetry in memory scores, highlighting the importance of considering lateralized hippocampal alterations in memory deficits.

A few studies evaluated the relationship between WADA memory scores and hippocampal volumes. Some reported a positive correlation between the hippocampal volumes and corresponding WADA memory scores (13). However, other studies failed to find such a correlation but instead reported a significant correlation between the asymmetries of these two measures (11, 12). These discrepancies may be attributed to various factors that could have affected the results of these studies. Small sample sizes, lack of stratification based on MRI findings (13) and variations in hippocampal volume measurement are among the key factors contributing to the divergent outcomes. Importantly, many earlier studies lacked standardized harmonization and normalization of hippocampal volumes with respect to total intracranial volume, which represents a critical methodological limitation (11–13). Failure to adjust for individual differences in brain size may introduce confounding variables and undermine the validity of conclusions drawn regarding the relationship between hippocampal volumes and memory scores.

In this study, the hippocampal volumes were harmonized and normalized based on the intracranial volume of gray and white matter, as this approach showed the strongest correlation with memory scores compared to adjustments made based on total intracranial volume or whole brain volume including the ventricles (20). In addition, this study is the first to evaluate the relationship between memory scores and hippocampal volumes using the Vol Brain software, an automated tool of volumetry measurement. This software demonstrated superior hippocampal segmentation accuracy compared to other publicly available automatic segmentation methods such as the FMRIB software library (FSL), Free Surfer, and statistical parametric mapping (SPM) (22–24).

In this study, we decided to stratify the results based on MRI findings, since we posited that the underlying pathological issues contributing to memory dysfunction would likely vary depending on the type and location of pathology. This decision proved to be sound, as major differences were observed in the correlation between absolute memory scores, WMA, and hippocampal volumetry between patients with MTS and those with normal MRI or other epileptogenic lesions.

MTS is radiologically characterized by reduced hippocampal volume, increased T2 signal intensity, and disturbed internal architecture (5, 25). Examination of neuropathological changes in the hippocampus of MTS patients revealed that neuronal cell loss in specific subfields and loss of granule cells in the dentate gyrus were key factors contributing to diminished declarative memory capacities (26, 27). In all MTS patients, we found that the WMA were concordant with the side of hippocampal atrophy. This finding supports the hypothesis that structural neuronal loss within the hippocampus correlates with memory deficits in these patients.

However, in patients with normal MRI findings or with structural lesions other than MTS, we did not find a correlation between harmonized normalized hippocampal volume and WADA memory scores, nor between WMA and hippocampal volume asymmetries. Nonetheless, in these patients, when the WMA difference exceeded 20% and in all patients who failed the WADA memory test on one side, lower memory scores were consistently seen on the side of the lesion or the side of the ictal onset zone. This suggests that lesions involving the mesial structures without directly affecting the hippocampus can disrupt memory pathways and lead to memory deficits such as by affecting the rhinal cortex or the parahippocampal gyrus, which are the afferent pathways to the hippocampus (28). In patients with normal MRI findings, the observed WMA may be attributed to lesions not evident on brain MRI affecting the mesial temporal structures such as a type I focal cortical dysplasia (29), subtypes of hippocampal sclerosis only affecting certain subfields (30–32) or disruption of the memory pathways due to frequent interictal discharges.

A particularly noteworthy and novel finding in our study is that, among patients with MTS, we were able to establish a harmonized normalized hippocampal volume threshold associated with a WADA memory score greater than 50% following injection into the contralateral carotid artery. Our ROC analysis identified a threshold of ≥28.94 units for this volume, with a sensitivity of 62.1% and a specificity of 100%. Therefore, in patients with MTS, a contralateral hippocampal volume ≥ 28.94 units was always associated with passing the WADA test, as reflected in a PPV of 100%. However, in these patients, a hippocampal volume < 28.94 units was associated with a relatively low NPV of 54.2%, indicating that volumetry is less reliable for detecting memory deficits in patients with smaller hippocampal volumes. This underscores that hippocampal volumetry performs exceptionally well in identifying normal WADA outcomes in patients with larger hippocampal volumes but is less sensitive in detecting WADA memory deficits, leading to a notable rate of false negatives. The observed false negatives, where patients with smaller hippocampal volumes passed the WADA test, likely result from a combination of factors. First, hippocampal volumetry, as a structural measure, does not account for functional or network-level compensatory mechanisms. For instance, patients with unilateral hippocampal sclerosis may rely on extrahippocampal regions, such as the parahippocampal gyrus or prefrontal cortex, to support memory performance during the WADA test, allowing them to pass despite significant atrophy. Second, individual differences in cognitive reserve may play a role. Patients with higher cognitive reserve may compensate for structural deficits, enabling them to perform memory tasks during the WADA test even with reduced hippocampal volume. This cognitive flexibility is not captured by volumetric assessments and could explain the discrepancies between hippocampal volume and WADA outcomes. Third, the inherent limitations of the WADA test itself should be considered. While it remains the clinical standard for assessing memory function, its sensitivity can be influenced by procedural nuances such as the dose of the anesthetic agent, the duration of hemispheric inactivation, and the degree of attentiveness to the tasks administered. However, it is important to emphasize that this threshold is specific to patients with MTS and does not apply to individuals with normal brain MRI or other types of epileptogenic lesions. While this finding provides preliminary insight into the association between hippocampal volume and memory performance during the WADA test in this specific cohort, it does not extend to prognosticating postsurgical outcomes. Further studies with larger sample sizes and direct analyses of postsurgical results are needed to validate and expand upon these findings. Our study possesses several strengths that enhance its reliability. Firstly, we employed a validated volumetry measurement software, ensuring accuracy and consistency in our measurements. Secondly, we obtained high quality epilepsy protocol MRI scans, interpreted by expert neuroradiologists, which minimizes the likelihood of misinterpretation or error. Thirdly, we stratified our analysis based on MRI findings, allowing for a more comprehensive understanding of the relationship between hippocampal volumes and memory function.

However, our study has several limitations. First, the sample size was relatively small, and the study was conducted retrospectively at a single center, potentially limiting the generalizability of our findings. Second, the MRI were acquired on different scanners with varying magnet strengths, a limitation addressed through harmonization. Additionally, due to financial constraints, we did not conduct preoperative and postoperative neuropsychological evaluations to assess changes in verbal and pictorial memory scores following surgery and their correlation with the WADA results.

Our findings highlight the importance of distinguishing between patients with MTS and those with normal MRI or other types of epileptogenic lesions when assessing hippocampal volumes and their correlation with WADA memory scores. In patients with MTS, hippocampal volumetry measurements could potentially serve as a viable alternative to the WADA test, particularly in cases where the WADA test cannot be conducted, such as individuals with cognitive impairments and those unable to cooperate. Moreover, in regions where access to WADA testing or fMRI is limited, hippocampal volumetry could provide a valuable alternative. However, the reliability of our results should be confirmed by future prospective studies exploring the relationship between hippocampal volumes and WADA memory scores. Such studies may demonstrate that non-invasive hippocampal volumetry is a viable replacement for the WADA test for patients with MTS who are candidates for temporal lobectomy.

Conversely, for patients with normal MRI or with epileptogenic lesions other than MTS, hippocampal volumetry cannot reliably predict memory deficits due to the lack of correlation between these measures. In such cases, it is imperative to conduct a WADA test or alternatively a fMRI, especially when planning resection of mesial temporal structures in the dominant hemisphere.

## Data Availability

The raw data supporting the conclusions of this article will be made available by the authors, without undue reservation.

## Footnotes

^1^ https://www.volbrain.net/

^2^ https://github.com/garciadias/Neuroharmony
