# Supplementary material for: Selective correlation of hippocampal volumes with WADA memory scores in mesial temporal sclerosis patients
Source: Front Neurol. 2025 Jan 24;16:1507846. doi: 10.3389/fneur.2025.1507846 (PMC11802426; doi:10.3389/fneur.2025.1507846)
Supplement: Supplementary file 1 [file Table_1.docx]

### **Supplementary Table:**

Patient Characteristics, MRI Findings, and WADA Memory Test Results in patients with Mesial Temporal Sclerosis

| Nb | Visual MRI analysis | Age | Gender | Failed WADA | Left hemisphere memory scores | Right hemisphere memory scores | Left harmonized normalized hippocampal volume | Right harmonized normalized hippocampal volume |
| --- | --- | --- | --- | --- | --- | --- | --- | --- |
| 1 | Bilateral MTS | 22.3 | M | No | 75.00% | 87.50% | 16.2 | 17.3 |
| 2 | Bilateral MTS | 26.2 | F | No | 68.75% | 56.25% | 26.0 | 24.4 |
| 1 | Right MTS | 30.8 | M | Right | 100.00% | 43.75% | 21.7 | 19.2 |
| 2 | Right MTS | 34.4 | M | Right | 87.50% | 37.50% | 31.4 | 20.9 |
| 3 | Right MTS | 17.5 | F | Right | 100.00% | 37.50% | 32.3 | 25.2 |
| 1 | Left MTS | 35.3 | M | Left | 25.00% | 75.00% | 23.0 | 32.3 |
| 2 | Left MTS | 22.1 | M | Left | 12.50% | 93.75% | 18.8 | 32.8 |
| 3 | Left MTS | 23.1 | M | Left | 31.25% | 100.00% | 23.1 | 32.1 |
| 4 | Left MTS | 27.7 | F | Left | 0.00% | 87.50% | 18.1 | 34.8 |
| 5 | Left MTS | 11.8 | M | No | 62.50% | 100.00% | 22.2 | 29.2 |
| 6 | Left MTS | 26.6 | M | No | 75.00% | 75.00% | 21.0 | 27.3 |
| 7 | Left MTS | 32.8 | F | No | 100.00% | 100.00% | 31.4 | 32.3 |
| 8 | Left MTS | 45.2 | F | Left | 25.00% | 87.50% | 28.7 | 33.9 |
| 9 | Left MTS | 30.6 | M | Left | 18.75% | 75.00% | 12.4 | 33.3 |
| 10 | Left MTS | 30.8 | M | No | 87.50% | 100.00% | 22.6 | 31.0 |
| 11 | Left MTS | 23.5 | F | No | 68.75% | 100.00% | 27.8 | 31.6 |
| 12 | Left MTS | 29.7 | F | No | 62.50% | 87.50% | 23.0 | 33.2 |
| 13 | Left MTS | 46.4 | F | Left | 0.00% | 81.25% | 26.1 | 37.3 |
| 14 | Left MTS | 28.5 | F | Left | 0.00% | 75.00% | 23.0 | 37.5 |
| 15 | Left MTS | 23.7 | F | Left | 0.00% | 100.00% | 26.4 | 33.1 |
| 16 | Left MTS | 20.0 | F | Left | 37.50% | 87.50% | 25.8 | 37.7 |
